# Incidence of Elevated Liver Enzyme Levels in Patients Receiving Remdesivir and Its Effective Factors

**DOI:** 10.34172/mejdd.2024.377

**Published:** 2024-04-30

**Authors:** Fatere Seyedalipour, Shabnam Alipour, Hamed Mehdinezhad, Rahim Akrami, Hoda Shirafkan

**Affiliations:** ^1^Clinical Research Development Unit of Ayatollah Rouhani Hospital, Babol University of Medical Sciences, Babol, Iran; ^2^Faculty of Pharmacy, Ayatollah Amoli branch, Islamic Azad University, Amol, Iran; ^3^Department of Internal Medicine, Rouhani Hospital, Babol University of Medical Sciences, Babol, Iran; ^4^School of Health, Mashhad University of Medical Sciences, Mashhad, Iran; ^5^Social Determinants of Health Research Center, Health Research Institute, Babol University of Medical Sciences, Babol, Iran

**Keywords:** Remdesivir, COVID-19, Aspartate transaminase, Alanine transaminase, Mortality, Adverse effects

## Abstract

**Background:**

Emergency use of remdesivir was approved for COVID-19 in some countries. Based on the promising results of remdesivir, the most common side effects were nausea, worsening respiratory failure, increased alanine aminotransferase levels, and constipation. The aim of this study was to determine the incidence of elevated liver enzymes in patients with COVID-19 receiving remdesivir.

**Methods:**

In this retrospective study, information was collected from patients’ files. The study population included patients with moderate to severe COVID-19 who were admitted to Rouhani Babol Hospital. For daily patient selection, the list of patients was extracted from the system, and based on the census, the patient file was selected. Data were analyzed using Stata 16.

**Results:**

620 patients suffering from moderate to severe COVID-19 were included in this study, 43% of whom were men. Of these patients, 120 were selected as the control group who did not receive remdesivir. The increase in liver enzymes in patients receiving remdesivir compared with the control, for alanine transaminase (ALT) and aspartate transaminase (AST), respectively, was 6.20 and 3.64 times, but it was not statistically significant for alkaline phosphatase (ALP). Also, the increase in bilirubin levels in patients receiving remdesivir was not statistically significant.

**Conclusion:**

The recipients of remdesivir had high liver enzymes, which is one of the possible side effects of this drug. The intensity of the enzymes was mild and moderate, and they were not dangerous to the health of any of the consumers. Deaths in patients with COVID-19 were not due to drug-induced liver complications but to other factors such as disease-related complications.

## Introduction

 COVID-19, a member of the coronavirus family, has had three major outbreaks to date, the most recent of which was the new (2019-nCoV) outbreak in 2019. On January 30, 2020, the World Health Organization (WHO) declared a global emergency due to a new coronavirus outbreak in Wuhan, a city in China’s Hubei province. Until January 29, 2022, more than 371 558 420 infected cases have been reported, which has led to the death of more than 5 670 967 people.^1^

 Based on the promising treatment results of remdesivir, emergency use of this drug was approved in some countries, including the United States, Japan, and Taiwan, in May 2020. The most commonly reported side effects of this drug were nausea, worsening respiratory failure, increased alanine aminotransferase (ALT) levels, and constipation.^2^ Complications of drug-induced liver injury can range from mild, transient, asymptomatic elevations in serum enzyme levels to acute liver failure leading to rapid death or the need for liver transplantation.^3^ Results of animal studies indicated no liver changes with remdesivir use,^4^ but a study published during the pandemic showed that patients treated with remdesivir were at risk of liver injury,^5^ and in some cases elevated liver enzymes and elevated bilirubin led to discontinuation of treatment with remdesivir.^6^ The results of clinical trials have also suggested that the incidence of side effects and biochemical disorders such as elevated liver enzymes, decreased hemoglobin and lymphocytes, and increased prothrombin time and blood glucose was higher in patients treated with remdesivir for 10 days than in patients treated for 5 days and more than three complications occurred in these individuals.^3^

 Due to the conflicting results and limited reports of side effects of remdesivir treatment on the liver and the lack of studies evaluating these side effects in Iran, studies of liver enzyme changes have been conducted in the treatment of patients with COVID-19. Due to their high incidence and the need to take remdesivir, this is a complication of concern that may provide good evidence for drug safety. The aim of this study was to determine the incidence of elevated liver enzymes in patients with COVID-19 receiving remdesivir.

## Materials and Methods

 In this retrospective study, information was collected from medical records. The study population consisted of 500 patients of both sexes (43% men) suffering from moderate to severe COVID-19 treated with remdesivir and hospitalized at Ayatollah Rouhani hospital in Babol. In addition to the main group, a control group was selected comprising 120 patients who were hospitalized for moderate to severe COVID-19 and did not receive remdesivir.

 The study was conducted by reviewing patients’ records and consulting with the treating physician and the ward nurses using a form designed by the project director. For daily patient selection, the patients’ list was extracted from the system based on the census, the patients’ records were selected, and the patients were enrolled in the study.The patients had a similar medication regimen.

 In this study, information collected for each patient included demographic data (age and sex), body mass index (BMI), inpatient ward, history of drug use and underlying diseases, clinical parameters recorded at baseline and results of serological tests, length of hospitalization in the intensive care unit (ICU), liver enzyme levels (ALT, aspartate transaminase [AST], alkaline phosphatase [ALP], and bilirubin levels), and treatment outcome during the treatment period.

 All COVID-19-positive patients aged 18 years and above were included in the study. Patients suffering from chronic liver diseases, alcoholism, hepatitis, pregnant women, and children (below 18 years) were excluded from the study.

 The severity of elevated liver enzymes was classified as follows^8^:

- Grade 1 (Mild) = 1.25-2.5 times the normal limit
- Grade 2 (Moderate) = 2.5-5 times the normal limit
- Grade 3 (Severe) = 5-10 times normal
- Grade 4 (life-threatening) = more than 10 times normal

Desired indicators such as length of hospital stay, ICU stay, need for supportive oxygen, and mortality in these groups were assessed.

 The data were collected by direct observation and based on patients’ records (except for the inpatient ward) and recorded in the checklist. The collected data were analyzed using Stata 16. Descriptive statistics were presented using mean and standard deviation (for quantitative data) and frequency and ratio (for qualitative data). Simple unconditional logistic regression and multiple logistic regression were performed to examine factors affecting patient mortality. Tests were used based on a significance level of 0.05.

## Results

 Personal and demographic characteristics information of those with COVID-19 into two groups receiving remdesivir and those not receiving remdesivir are provided in Table 1.

**Table 1:** The personal and demographic characteristics of patients with COVID-19 who received remdesivir compared with the control group

**Variable**		**Remdesivir drug group**	**Control group**
		**Mean (SD)/No. (%)**	**Mean (SD)/No. (%)**
Age (years)		52 (16)	59 (17)
Sex	Male	214 (43)	55 (42)
	Female	286 (57)	75 (58)
Body mass index (kg/m^2^)		28 (4)	27 (4)
Underlying disease			
	No	380 (76)	89 (68)
	Blood pressure	45 (9)	15 (12)
	Diabetes	55 (11)	19 (15)
	Hypothyroidism	20 (4)	7 (5)
Use of oxygen	No	60 (12)	25 (19)
	Yes	440 (88)	105 (81)
Use of mechanical ventilation	No	444 (89)	111 (85)
	Yes	56 (11)	19 (15)
Hospitalization in the ICU	No	447 (89)	114 (88)
	Yes	53 (11)	16 (12)
Mortality	No	479 (96)	119 (92)
	Yes	21 (4)	11 (8)
		Median (25th percentile - 75th percentile)	Median (25th percentile - 75th percentile)
Duration of hospitalization (days)		7 (10-5)	7 (9-5)

 In this study, the effect of taking remdesivir on the potential to increase important enzymes such as ALT, AST, ALP and bilirubin was investigated. The required analysis was performed, and the results are shown in the tables below. In addition, the association of demographic, clinical, and underlying diseases with the increase in the above enzymes in patients with COVID-19 was investigated and presented.

 Considering that ALT enzyme elevation is defined as a three-level variable without grade 1 increase and grade 2 increase, logistic rank regression analysis was used to investigate the association between this variable and remdesivir. According to the results of the subjects who received the remdesivir drug, the probability of an increase in the enzyme (second and first degree) was 6.20 times higher compared to no increase (Table 2).

**Table 2:** Association between remdesivir use and ALT enzyme elevation in patients with COVID-19 at Babol University of Medical Sciences

**Univariate logistic regression model**			Multivariate logistic regression model*		
**Odds ratio**	***P*** ** value**	**95% confidence interval**	**Odds ratio**	***P*** ** value**	**95% confidence interval**
5.30	< 0.001	3.39-8.27	6.20	< 0.001	3.90-9.86

* Age, duration of hospitalization, duration of ICU hospitalization, sex, use of mechanical ventilation, use of oxygen and hospitalization in ICU were adjusted. Then, the backward stepwise logistic regression model was used.

 Considering that AST enzyme elevation is defined as a three-level variable without grade 1 and grade 2 increase, logistic rank regression analysis was used to investigate the association between this variable and remdesivir. According to the results of the subjects who received the remdesivir drug, the probability of an increase in the enzyme (second and first degree) was 3.64 times higher compared with no increase (Table 3).

**Table 3:** Association between remdesivir use and AST enzyme elevation in patients with COVID-19 at Babol University of Medical Sciences

**Univariate rank logistic regression model**			Multivariate logistic regression model*		
**Odds ratio**	***P*** ** value**	**95% confidence interval**	**Odds ratio**	***P*** ** value**	**95% confidence interval**
3.11	< 0.001	1.90-5.08	3.64	< 0.001	2.18-6.07

* Age, duration of hospitalization, duration of ICU hospitalization, sex, use of mechanical ventilation, use of oxygen and hospitalization in ICU were adjusted. Then, the backward stepwise logistic regression model was used.

 The regression model indicated that in univariate analysis, there was no significant association between the use of remdesivir in patients with COVID-19 and ALP enzyme increase. After controlling for confounding variables, the results suggested that individuals who received remdesivir had no significant difference in enzyme compared with the control group.

 The regression model indicated that in univariate analysis, there was no significant association between the use of remdesivir in patients with COVID-19 and total bilirubin increase. After controlling for confounding variables, the results suggested that individuals who received remdesivir had no significant difference compared with the control group.

 The severity of elevated liver enzymes in patients with COVID-19 receiving remdesivir compared with the control group was 6.20-fold for ALT, 3.59-fold for AST, and not statistically significant for ALP and total bilirubin.

 For the association between elevated liver enzyme levels and sex in patients with COVID-19 receiving remdesivir, male sex increases the risk 1.56-fold for ALT, 1.7-fold for ALP, and 1.86-fold for total bilirubin. There was no association between sex and elevated AST enzymes.

 For the association between the number of doses of remdesivir and elevated liver enzymes in patients with COVID-19 receiving remdesivir, the risk for elevated AST enzymes for every one dose of the drug was 17%. There was no correlation between the number of doses of remdesivir received and increases in the enzymes ALT, ALP, and total bilirubin.

 During hospitalization, the patients with COVID-19 receiving remdesivir increased the risk of ALT by 6%, AST by 10%, ALP by 13%, and total bilirubin by 14% for each day of hospitalization. The patients with COVID-19 receiving remdesivir in the ICU had a one-day increase in the risk of enzyme elevation: AST 8%, ALP 11%, and total bilirubin 12%. There was no association between length of ICU stay and elevated ALT enzymes.

 For the association between the use of mechanical ventilation and elevated liver enzymes in patients with COVID-19 receiving remdesivir, the risk of increase is 2.24-fold for AST, 2.35-fold for ALP, and 3.21-fold for total bilirubin. There was no association between the use of mechanical ventilation and elevated ALP enzymes.

 There was no association between elevated liver enzymes and age, BMI, use of oxygen, underlying disease, and mortality in patients with COVID-19 receiving remdesivir.

 Treatment was not discontinued in any of the patients with COVID-19 who received remdesivir and had elevated liver enzymes. Hepatic complications were reversible in patients with COVID-19 who received remdesivir and had elevated liver enzymes.

## Discussion

 The current study showed that the patients receiving remdesivir were at greater risk of elevated liver enzyme levels (especially ALT and AST) compared with the control group. Prior studies on patients with COVID-19 have reported that 37%–69% of such patients experience at least one abnormal ALT and AST during hospital admission.^7^

 Regarding the association between elevated liver enzyme levels and sex in patients with COVID-19 receiving remdesivir, males were at greater risk of elevated liver enzyme levels compared with females. The distribution of remdesivir drug is more in the liver and kidney organs and to a lesser extent in the heart and lungs.^9^ Anatomical and physiological differences in male and female sex can affect the pharmacokinetics and pharmacodynamics of the drugs in the body.^10^

 Aging and BMI increase result in a decrease in the amount of water in the body, leading to an increase in fat tissue. Thus, drugs that dissolve in water reach higher concentrations and drugs that dissolve in fat accumulate more. Also, in aging people, kidneys are less able to excrete drugs into the urine, and the liver is less able to metabolize many drugs.^11^ The current study showed that in patients with COVID-19 receiving remdesivir, there was no correlation between elevated liver enzymes and patients’ age. Similarly, there was no association between elevated liver enzymes and BMI.

 Regarding the association between the number of doses of remdesivir and elevated liver enzymes in patients with COVID-19 receiving remdesivir, there was no correlation between the number of doses of remdesivir received and increases in the enzymes ALT, ALP, and total bilirubin, but patients who received more doses of remdesivir had elevated AST. The recommended dosage of remdesivir for patients with COVID-19 is a single loading dose of 200 mg followed by once-daily maintenance doses of 100 mg via intravenous infusion over 30 to 120 minutes for 5-10 days. The toxicity caused by acute exposure to remdesivir can only be roughly estimated from the currently reported adverse effects.^12^

 During hospitalization, patients with COVID-19 receiving remdesivir were at greater risk of elevated liver enzyme levels for each day of hospitalization compared with the control group. COVID-19 patients receiving remdesivir in the ICU are at risk of enzyme elevation of AST, ALP and total bilirubin. There is no association between the length of ICU stay and elevated ALT enzymes. It is conclusive whether patients receiving remdesivir for more days (ICU and non-ICU) would have had more AEs.^12^

 There was no correlation between the use of oxygen and elevated liver enzymes in patients with COVID-19 receiving remdesivir. Prior studies on patients with COVID-19 have reported that improvement in oxygen-support status was observed in 68% of patients.^13^

 In the association between the use of mechanical ventilation and elevated liver enzymes in patients with COVID-19 receiving remdesivir, mechanically ventilated patients are at greater risk of elevated liver enzyme levels, but there was no association between the use of mechanical ventilation and elevated ALP enzymes. Prior studies on patients with COVID-19 have reported that remdesivir exerts a beneficial effect in terms of survival in such patients undergoing mechanical ventilation.^14^ In COVID-19 patients receiving remdesivir both the benefits and the risks need to be evaluated.

 There was no association between elevated liver enzymes and mortality in patients with COVID-19 receiving remdesivir. Also, There was no association between underlying disease and elevated liver enzymes in such patients. To avoid any severe adverse effects, we suggest monitoring liver function tests and renal function tests for patients, especially those with hepatic and renal impairments.

 The present study has some limitations: Our investigation was a single-center study. Other limitations of our investigation were different viral strains and lack of COVID-19 control patients who did not receive remdesivir. Hence, more extensive studies with detailed information about the type of infecting strains are necessary to separate the patients and compare them.

## Conclusion

 The results of this study showed that patients with COVID-19 who received remdesivir had elevated liver enzymes, a possible side effect of the drug. The severity of the elevated enzymes was mild and moderate, and they were not dangerous or hazardous to the health of any of the patients taking the drug. Mortality in patients with COVID-19 was not due to liver complications from the drug but was due to other factors such as disease-related complications and other factors. In addition, the liver complications were reversible and not permanent. There was no association between age, BMI, and underlying diseases. This drug is suitable for elderly patients with underlying diseases above the standard BMI. However, care should be taken when prescribing or taking any medication. This information will facilitate the establishment of treatment protocols for patients with COVID-19.
